# Development, Relative Validity and Reproducibility of the Aus-SDS (Australian Short Dietary Screener) in Adults Aged 70 Years and above

**DOI:** 10.3390/nu12051436

**Published:** 2020-05-15

**Authors:** Adelle M. Gadowski, Tracy A. McCaffrey, Stephane Heritier, Andrea J. Curtis, Natalie Nanayakkara, Sophia Zoungas, Alice J. Owen

**Affiliations:** 1 School of Public Health and Preventive Medicine, Monash University, Melbourne VIC 3004, Australia; adelle.gadowski@monash.edu (A.M.G.); Stephane.Heritier@monash.edu (S.H.); Andrea.Curtis@monash.edu (A.J.C.); Natalie.Nanayakkara@monash.edu (N.N.); Sophia.Zoungas@monash.edu (S.Z.); 2 Department of Nutrition, Dietetics and Food, Monash University, Clayton 3168, Australia; Tracy.Mccaffrey@monash.edu

**Keywords:** diet, dietary intake, dietary screener, brief questionnaire, food group, older adults, relative validity, validity

## Abstract

The aim of this study was to assess the relative validity and reproducibility of a six-item Australian Short Dietary Screener (Aus-SDS). The Aus-SDS assessed the daily intake of core food groups (vegetables, fruits, legumes and beans, cereals, protein sources and dairy sources) in 100 Australians (52 males and 48 females) aged ≥70 years. Relative validity was assessed by comparing intakes from the Aus-SDS1 with an average of three 24-h recalls (24-HRs), and reproducibility using two administrations of the Aus-SDS (Aus-SDS1 and Aus-SDS2). Cohen’s kappa statistic between the Aus-SDS1 and 24-HRs showed moderate to good agreement, ranging from 0.44 for fruits and dairy to 0.64 for protein. There was poor agreement for legume intake (0.12). Bland–Altman plots demonstrated acceptable limits of agreement between the Aus-SDS1 and 24-HRs for all food groups. Median intakes obtained from Aus-SDS1 and Aus-SDS2 did not differ. For all food groups, Cohen’s kappa statistic ranged from 0.68 to 0.89, indicating acceptable agreement between the Aus-SDS1 and Aus-SDS2. Spearman’s correlation coefficient between Aus-SDS1 and 24-HRs across all food groups ranged from 0.64 for fruit to 0.83 for protein. We found the Aus-SDS to be a useful tool in assessing daily intake of core food groups in this population.

## 1. Introduction

The burden of chronic diseases, particularly cardiovascular disease, is steadily increasing, largely due to increasing population age [1]. More than 1 in 4 (29%) Australians aged ≥65 years have at least three chronic diseases, compared with just 2.4% of those aged under 45 years [2]. Identifying ways to improve overall morbidity and mortality with an aging population is crucial. The World Health Organization (WHO) have reported both suboptimal dietary behaviours and physical inactivity to be a major modifiable determinant of chronic diseases [3]. The Global Burden of Disease study reported that 11 million deaths and 255 million disability-adjusted life-years (DALYs) across 195 countries were attributable to suboptimal dietary behaviours [4]. Dietary intake is impacted by biological, social and economic factors [5]. Older adults tend to differ in dietary patterns compared to young adults, and are at greater risk of malnutrition (undernutrition and overnutrition). At least 10% of community living adults aged ≥65 years are malnourished with a further 35% at risk of suboptimal nutrition [6]. Impacts of malnutrition can result in adverse effects on body composition, function and clinical outcomes [7].

In recent years, nutritional epidemiological research has increasingly taken ‘whole diet’ (e.g., intake of food groups or dietary patterns) as opposed to single nutrient approaches, given that nutrients are not consumed in isolation [8]. This has translational benefits as public health strategies to improve diet are usually targeted to specific food groups and whole diets [9], and healthy diet patterns are associated with positive health outcomes [10]. Health bodies around the world, including in Australia, have developed evidence-based dietary guidelines to improve quality of life and reduce chronic disease risk across the lifespan [11]. However, despite evidence of the importance of healthy dietary behaviours for reducing the burden of disease [12], as well as a greater adherence to published dietary guidelines to be associated with an overall reduced risk of mortality in older Australians [13], tools to evaluate the dietary intake of core food groups in older adults are limited [14].

Detailed dietary intake assessment in large-scale epidemiologic studies is challenging, as tools designed to capture dietary intakes are often expensive, time-consuming and burdensome on both participants and researchers [15]. Factors related to age such as declines in cognitive function and impaired hearing or vision, may limit an individual’s ability to provide accurate information regarding their dietary behaviours [16]. Being more cost-effective and less labor intensive, food frequency questionnaires (FFQs) and dietary screeners are more often utilized in nutritional epidemiological research. However, dietary intake behaviours are known to change with age [5] and, therefore, dietary assessment tools need to be developed and validated in the specific population groups to which they will be applied.

Few screening tools have been developed to assess dietary intake in older populations, and these tools generally focus on a specific dietary component; such as fruit and vegetables [17,18], fat [19], calcium [20] or screeners addressing both nutrients and dietary patterns [21,22]. To our knowledge, no short screening tools examining all core food groups (vegetables, fruits, legumes and beans, cereals, protein sources and dairy sources) have been developed and validated specifically for use in the older Australian population. Such a tool would provide a simplified, less burdensome approach for older participants and researchers in large-scale epidemiological studies examining diet and health in older age. Therefore, this study aims to assess the relative validity and reproducibility of the Australian Short Dietary Screener (Aus-SDS), in a population aged ≥70 years, a tool designed to capture usual intake of core food groups in accordance with the Australian Dietary Guidelines [11].

## 2. Materials and Methods

### 2.1. Study Design

**Figure 1:**
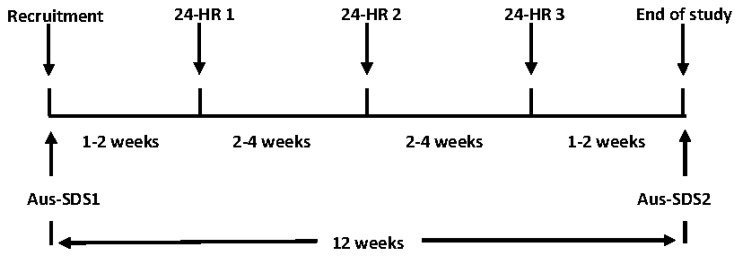
Timeline of study. Three 24-h recalls (24-HRs) were administered at intervals of 2–4 weeks, commencing 1–2 weeks after the first Australian Short Dietary Screener (Aus-SDS1) was administered. Aus-SDS2 was administered 1–2 weeks after completion of the final 24-HR. Total study duration was 12 weeks.

The study was of a 12-week duration, conducted between March and June 2019. Participants were recruited via postal invitation and provided written informed consent prior to study participation. The first Aus-SDS (Aus-SDS1) and a lifestyle questionnaire were delivered via post. The lifestyle questionnaire included questions on self-reported participant demographics (date of birth, gender, height, weight, highest education level, income, household composition), medical history (presence of heart disease, type 1 and 2 diabetes, depression, cancer, coeliac disease, irritable/inflammatory bowel disease, Crohn’s disease, kidney disease, high cholesterol and food intolerances), nutritionist or dietitian consultation in the previous year, smoking history, alcohol use and use of cholesterol-lowering, blood pressure-lowering or glucose-lowering medication. One to two weeks after the completed Aus-SDS1 and lifestyle questionnaires were returned via post, participants completed their first telephone interviewer-administered 24-HR (24-h dietary recall). During the study period, three 24-HRs were collected from each participant at two- to four- week intervals. The second Aus-SDS (Aus-SDS2) was administered one to two weeks after completion of the third 24-HR. The study design is shown in Figure 1. 

### 2.2. Study Population

The study population was identified from an existing database of older adults who had previously registered an interest in participating in healthy ageing research. Registered participants were community-living adults, aged 70 years and above, and able to read and write in English. Of the randomly selected 400 people invited, 137 consented to take part in the study and complete data was obtained from 100 participants (52 males and 48 females) (Supplementary Figure S1). Bland and Altman recommend a sample size of 100 to be adequate for comparison of agreement between two methods [23]. 

### 2.3. Ethics Statement

This study was approved by the Monash University Human Research Ethics Committee (MUHREC) (Project ID 13963), and was conducted in a manner consistent with the Declaration of Helsinki. 

### 2.4. Diet Screening Tool for Older People (Australian Short Dietary Screener, Aus-SDS)

The Aus-SDS (Supplementary Figure S2) was developed based on the questionnaire structure proposed by Cook et al., which used single questions to measure fruit and vegetable intake during the past year [18]. The food groups in Aus-SDS were selected based on the core food groups identified in the Australian Dietary Guidelines and Australian Guide to Healthy Eating (AGHE) [11]. The Aus-SDS was divided into six groups covering vegetables (dark green or cruciferous/brassica, root/tubular/bulb vegetables and other vegetables), fruits (pome, citrus, stone, tropical, berries and other fruits), cereal (grain based foods, mostly wholegrain and/or high cereal-fibre varieties), legume and beans, protein sources (lean meat and poultry, fish, eggs, tofu, nuts and seeds) and dairy sources (milk, yoghurt, cheese and alternatives). As legumes and beans contribute to both vegetable and protein food groups, with one serve of vegetables (75 g) and one serve of protein (150 g), we separated legume and beans to avoid the possibility of participants overestimating intake. 

The frequency of consumption categories were adapted from previous national surveys [18] and remained consistent for each food group. Participants were asked to think about their usual intake over the past 12 months and asked: ‘How many serves of each food group did you usually eat per day?’. Separate responses were included for each of the six food groups. The Aus-SDS used 12 response options; never, less than 1 serve per month, 1 to 3 serves per month, 1 serve per week, 2 serves per week, 3–4 serves per week, 5–6 serves per week, 1 serves per day, 2 serves per day, 3 serves per day, 4 serves per day, and 5 or more serves per day. A brief description of what constituted a serve for each food group, based on the AGHE descriptions [11], was provided; for example, participants were asked to count 1 serve of vegetables as either; 1 cup of raw salad vegetables, or ½ cup of cooked salad vegetables. Examples of food items under each food group were provided to avoid duplication or inclusion of possible discretionary foods (foods or drinks that do not fit into the core food groups as they are too high in saturated fat and/or added sugars, added salt or alcohol and low in fibre [11]) or foods that do not contribute to the core food group. The Aus-SDS is available as online supplemental material (Supplementary Figure S2). In development-phase pilot testing of the Aus-SDS, it took approximately 10 min for older adults to complete.

### 2.5. Multiple Pass 24-h Recall Dietary Assessment

To account for day-to-day variation in dietary intake, three repeated 24-HRs were obtained at two to four week intervals. We included two week-days and one weekend day to capture any variability in intake that may occur [24]. Each 24-HR was administered by a trained registered nutritionist using an established multiple pass method [25]. Full details of the multiple pass recall method have been published elsewhere [26]. Briefly, 24-HRs were performed using a 5-step approach: stage 1 involved a quick recall to collect all foods and beverages consumed in the prior 24-h; stage 2 involved recall prompts for forgotten foods or consumption occasions; stage 3 recalled the time and location of eating occasions for all foods; stage 4 involved a more detailed cycle, including the description of foods, amount, cooking method, condiment additions and brands, if known. Stage 5 involved a final enquiry to recall any forgotten foods. Participants were also provided with a hard copy of the Australian Health Survey Food Model Booklet [27] to assist in estimating serving size during the 24-HRs. 

### 2.6. Data Analysis

To be included in analyses, participants had to complete both Aus-SDS questionnaires and three 24-HRs (excluded *n* = 10) (Supplementary Figure S1). Demographic characteristics of the study population were explored using descriptive statistics. Continuous variables were summarised using mean and standard deviation (SD) or median and interquartile range (IQR) for skewed data. Categorical variables were described as percentages. Body mass index (BMI) was calculated as weight (kg) divided by height squared (m^2^). Education status was categorized into two groups; high school or less, or more than high school (certificate/diploma or tertiary qualification). Smoking status and alcohol use was categorized as current, former or never. Annual income was self-reported and categorized based on the median result of <$35,000 and ≥$35,000. Household composition was categories into two groups; single person household (living alone) or group household (living with spouse/partner, other family members or friends).

All dietary intake data from the 24-HRs were entered into Foodworks (Version 9, Australia: Xyris Software). Data was then converted to daily food group servings equivalents using reference servings from the Australian Dietary Guidelines [11]. All 24-HR data entry and disaggregation was performed by a registered nutritionist and field worker. Food items were coded using the Food Standards Australia New Zealand AUSNUT 2011-13 database [28]. Where composite dishes were reported, participants were asked to provide ingredients. Where ingredients of composite dishes were not known e.g., branded/store bought items, or where composite dishes were so diverse that no relevant main category could be identified, the following applied [29]: foods containing more than one distinct ingredient were disaggregated into 11 categories; vegetable, fruit, cereal, legume and beans, protein sources, dairy, discretionary foods [30], condiments, alcoholic beverages, non-alcoholic beverages and fats and oils. Daily intake of core food groups from the three 24-HRs was then averaged to create an overall average daily intake for each participant. For data entry verification, a random sample of 20% of the Aus-SDS1 and Aus-SDS2 as well as 10% of 24-HRs were double entered.

Data from Aus-SDS1 and Aus-SDS2 was converted to a daily equivalent frequency (DEF) for each food group using the following reference adapted from the Victorian Cancer Council FFQ User Guide [31] to accommodate the different response categories in the Aus-SDS: never; 0 DEF, less than 1 serve per month; 0.02 DEF, 1 to 3 serves per month; 0.07 DEF, 1 serve per week; 0.14 DEF, 2 serves per week; 0.28 DEF, 3–4 serves per week; 0.50 DEF, 5–6 serves per week; 0.78 DEF, 1 serve per day; 1 DEF, 2 serves per day; 2 DEF, 3 serves per day; 3 DEF, 4 serves per day; 4 DEF, and 5 or more serves per day; 5 DEF. 

Relative validity of the Aus-SDS was assessed by comparing results from the Aus-SDS1 and the average of three 24-HRs as recommended by Bland and Altman [23]. The Bland–Altman plots graphically display the difference between X = Aus-SDS1 and Y = average of three 24-HRs vs. their average (X + Y)/2 and the limits of agreement between the two methods. We validated the Aus-SDS using the first administration to reduce any impact of previous exposure to the questionnaire. The reproducibility of the Aus-SDS was assessed by examining mean difference (bias) and 95% limits of agreement (LOA) (the mean difference ± 2 standard deviations for the difference between the two measurements) between the Aus-SDS1 and Aus-SDS2. A two-sided significance level of <0.05 was considered statistically significant. The ability for the Aus-SDS to accurately rank participants within quartiles was assessed using Cohen’s kappa statistic, including 95% confidence intervals, to provide measures of both consistency and agreement between repeat administrations. Spearman rank correlation coefficients was used to measure the direction and strength of the monotonic relationship between methods. The 95% confidence intervals were reported using Fisher transformation for Spearman correlation coefficient, and bias corrected bootstrap (500 repetitions) for Cohen’s kappa. Statistical analyses were performed by a registered nutritionist and statistician using SPSS 24 (IBM Corporation, Chicago, IL, USA) and Stata statistical software version 15 (StataCorp, TX, USA).

## 3. Results

### 3.1. Sociodemographic, Medical and Anthropometrics Characteristics

Ten participants were excluded from analyses due to having incomplete data (mean (standard deviation, SD) age 76.3 (3.5) years and 30% male (Supplementary Figure S1)). In total, 100 participants (52 men and 48 women) completed all aspects of the study and were included for analyses. The characteristics of the study population are described in Table 1. The mean age was 76.8 years (SD 4.5) and mean BMI was 26.7 kg/m^2^ (SD 3.8). Most participants had completed a tertiary level education (*n* = 65). The majority of participants were either former smokers (*n* = 49) or had never smoked (*n* = 49), and most were current alcohol consumers (*n* = 80). The majority of participants (*n* = 80) reported an annual income of less than $35,000 and lived in a multi-person household (*n* = 74) (living with spouse/partner, family member or friend). Approximately half of participants reported having high cholesterol, and 63 reported taking cholesterol-lowering medication.

### 3.2. Dietary Intake

Overall the Aus-SDS1 reported slightly higher median (IQR) intake of fruit, legume and dairy intake compared to the 24-HRs (Table 2). There were similar median intakes across all food groups between the Aus-SDS1 and Aus-SDS2.

### 3.3. Relative Validity of the Aus-SDS

**Figure 2:**
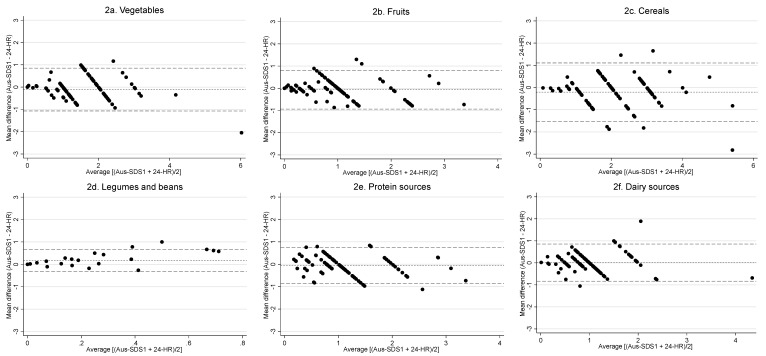
Bland–Altman plot showing the agreement between core food groups (vegetables, fruits, legumes and beans, cereals, protein sources and dairy sources) (serves/day) derived from the Australian Short Dietary Screener (Aus-SDS), and 24-h recalls (24-HR). Mean difference (bias) is represented by the dotted line, the upper and lower limits of agreement (LOA) by the longer broken lines.

The Bland-Altman plots are shown in Figure 2a–f for all core food groups. The plots generally show good agreement with no structure indicative of bias, and a small number of observations beyond the 95% LOA compatible with the 5% expected rate [32], with a few outliers noted with vegetable, cereal and dairy intake. Details on the distribution of intake of food groups assessed through the Aus-SDS1 and average of the three 24-HRs are presented in Table 3. Kappa coefficient ranged from 0.44 to 0.64 across all food groups, with the exception of legumes and beans (Figure 2d), indicating moderate to good agreement. Overall the mean differences across all food groups were similar between the Aus-SDS1 and 24-HRs, with the Aus-SDS1 only marginally reporting a lower intake for all food groups apart from legume and dairy.

### 3.4. Reproducibility of the Aus-SDS

Table 4 illustrates the comparison of results from the Aus-SDS1 vs. Aus-SDS2 through Spearman’s correlation coefficients and Cohen’s kappa statistic. Overall, the two administrations of the Aus-SDS reported similar intakes across all food groups. Mean differences between the Aus-SDS1 and Aus-SDS2 were minimal, ranging from 0.01 (95% confidence interval (CI) −0.02, 0.04) for legume intake to 0.10 (95% CI 0.03, 0.16) for fruit intake. Spearman’s correlation coefficient ranged from 0.84 for cereal, 0.86 for legume and protein, 0.91 for vegetable and dairy to 0.95 for fruit intake. Kappa coefficient ranged from 0.68 to 0.89 across all food groups, indicating good to very good agreement.

## 4. Discussion

This study evaluated the relative validity and reproducibility of the Aus-SDS. To our knowledge, this is the first screening tool examining all core food groups (vegetables, fruits, legumes and beans, cereals, protein sources and dairy sources) in an older Australian population. Data analyses confirmed good relative validity and reproducibility of the dietary screening tool, designed to capture the usual intake of core foods in an Australian population aged ≥70 years. Intakes of vegetables, fruits, cereal and protein were slightly underestimated using the Aus-SDS1 compared to 24-HRs, whereas dairy intake was similar in both Aus-SDS1 and 24-HRs and legume intake was slightly overestimated using the Aus-SDS compared with 24-HRs. The advantages of a short dietary screener are seen in terms of low participant and researcher burden, and may be a cost-effective approach where adherence to core food groups is the outcome of interest. Thereby enabling its use in large scale epidemiological studies examining intake of core food groups in an older population.

Bland Altman analyses were used to illustrate the agreement between the Aus-SDS1 and the 24-HRs by comparing the differences between the tools against the mean of the two tools. When the mean of differences is closer to zero it indicates a narrower agreement interval and, therefore, a better agreement between the two tools [23]. Overall the mean differences in daily food group intake between the Aus-SDS1 and 24-HRs were similar across all food groups and, therefore, the Aus-SDS showed a good relative validity compared to the 24-HRs. As an example, the Aus-SDS underestimated vegetable intake by 8 g, and fruit intake by 9 g compared to 24-HRs. These differences are within acceptable limits, given that the 95% margin of error for grams of usual intake in Australians aged 71 years or over in 2011–2012 was reported to be 23.1 g for vegetables and legumes, and 23.8 g for fruit [33]. However what constitutes a meaningful change in dietary behaviours needs to be the subject of further research [34].

Masson et al. have suggested a correlation coefficient over 0.50 to be acceptable in dietary validation studies [35], with our study well above this threshold (ranging from 0.64 for fruit to 0.84 for cereal), and comparable with that of similar research. A study conducted in Canada, validating a short diet questionnaire against three 24-HRs in older adults reported Spearman correlation coefficients of 0.45 for fruit and vegetables [36]. An Australian study validating a short diet questionnaire on vegetable and fruit intake reported Spearman correlation coefficients of 0.86 for fruit and 0.52 for vegetables [18]. A study of older adults in Italy, reported Spearman correlation coefficients between a FFQ and 24-HRs ranging up to 0.85 for food groups [37].

Masson et al. also suggested kappa statistic values above 0.40 to be desirable for false-negative associations in validation studies to be minimized [35]. A moderate kappa agreement was observed for vegetable, fruit, cereal and dairy intake (kappa 0.44 to 0.58). A good kappa agreement was observed with protein intake (0.64). We observed a poor kappa agreement with legume intake (0.12), however this may be influenced by the difficulties of capturing foods that are infrequently eaten, specifically when assessing dietary intake over a 24 h period as done through 24-HRs rather than dietary screeners, FFQs or diet diaries reporting intake frequency over a longer period of time [15].

The reproducibility of the Aus-SDS as shown through Spearman correlation coefficient is similar to that of previous research. A study conducted in Singapore (age range 18–79 years), validating a short 37-item dietary screener measuring food groups included in international healthy eating indices (Alternate Healthy Eating Index 2010, Alternate Mediterranean Diet and Dietary Approaches to Stop Hypertension), with a similar frequency reporting scale as the Aus-SDS reported correlation coefficients of 0.63 for vegetables, 0.52 for fruit, 0.68 for cereal and 0.65 for dairy fat [38]. Similarly, a study of 100 Australian men and women (mean age 60 years) examining the reproducibility of a 135-item FFQ on nutrients and food groups (18 food groups in total) reported kappa values ranging from 0.37 for bread and cereals to 0.71 for low-fat dairy and green leafy vegetables [39]. A possible explanation for the high correlations in our study was the short interval between the Aus-SDS1 and Aus-SDS2, which may indicate the possibility that participants may have recalled their previous response.

This study has several advantages. The simplicity in design and postal administration of the Aus-SDS in measuring the intake of core food groups shows great advantage in a tool specifically designed for older populations, given that it is well known for older populations to respond more accurately in simplified questionnaires [40]. The design of the Aus-SDS may also offer greater time and cost-efficiency for researchers in large-scale epidemiological research. Our study sample specifically targeted older community-dwelling Australians, with a good gender representation and completion rate from those consenting to take part (73% completion rate).

Our sample size was based on multiple dietary validation studies that reported similar sample sizes [18,41]. Intake of discretionary foods was not collected by the Aus-SDS, as the focus of this study was to examine adherence to healthy dietary patterns which are well documented in positive health outcomes [9]. The study design used self-reported dietary intake; nevertheless, previous data suggest approximately 90% of self-reported information to be accurate, and questionnaires to be a reliable way of gathering data regarding patient self-care practices [42]. Participation bias is possible as participants were recruited from an existing database of older adults expressing interest in being involved in future research, and health literate individuals may be more likely to participate in healthy ageing research [43]. Both the Aus-SDS and 24-HRs have similar sources of error, in that they are both dependent upon the participant’s accuracy and memory and may be subject to bias due to underestimating or overestimating actual dietary intake. Self-reported dietary intake also has the possibility to be influenced by desirability bias, a type of responder bias where participants may over- or underestimate their dietary intake to that which they perceive may be more favourably viewed, a common bias identified among older adults [44].

A number of suggestions can be made to improve outcomes in future relative validity testing. Despite legumes being included as a separate food group in the Aus-SDS, participants were not specifically instructed to exclude legume intake for vegetable and/or protein food groups. Therefore, it is possible that participants may have overestimated vegetable and/or protein intake. Overall, 86 participants reported no intake of legume and beans through 24-HR dietary assessment, compared with 9 participants using the Aus-SDS1. This is a common limitation with tools measuring ‘actual’ intake as done though the 24-HR, as it is dependent on the participant consuming that food in the prior 24 h. Whereas tools measuring ‘usual’ intake such as the Aus-SDS, require participants to recall their usual intake over the prior 12 months [24]. These differences highlight the strength of the Aus-SDS and limitation of the multiple 24-HRs in assessing ‘usual’ intake. Further research would be required to address this, specifically through additional repeat measures of the 24-HRs to reflect accurate usual intake as well as larger population size.

Seasonal variation in dietary intake may not have been captured with the 24-HR administration as the study duration of 12 weeks occurred during autumn and winter. However, previous research indicates there are minimal seasonal differences in dietary patterns and overall diet quality [45], with similar research reporting agreement between repeated measures of dietary intake ranging from 15 days to several years [46,47].

Finally, our study population was predominantly Australian-born and English-speaking which may limit the generalizability of our findings.

## 5. Conclusions

This study evaluated the relative validity and reproducibility of a six-item dietary screener developed specifically for measuring daily intake of core food groups in an Australian population aged ≥70 years. This may be particularly useful in large scale epidemiological studies, where dietary intake is not a primary measure. The results in the present study suggest the Aus-SDS to be a valid tool in assessing adherence to core food groups in accordance with the Australian Dietary Guidelines. Further validation of the Aus-SDS, including against biomarkers, would be beneficial in other populations including in individuals with English as a second language, those below 70 years of age, in populations with specific medical comorbidities and also the potential for the Aus-SDS to be adapted to international populations with similar interests in intake of the same core food groups.

## Figures and Tables

**Table 1:** Characteristics of study population.

Variables		Participant (*n* = 100)	
		Mean	SD
Age (years)		76.8	4.5
Height, cm		168.1	10.2
Weight, kg		75.9	14.2
BMI (kg/m^2^)		26.7	3.8
		**Number**	
Gender			
	Male	52	
	Female	48	
Education			
	High school or less	35	
	More than high school	65	
Smoking status			
	Current	2	
	Former	49	
	Never	49	
Alcohol use			
	Current	80	
	Former	7	
	Never	13	
Annual income			
	<$35,000	59	
	≥$35,000	33	
	Prefer not to say	8	
Household composition			
	Single person household	26	
	Multi-person household	74	
Medical history			
	Heart disease	27	
	Diabetes	14	
	High cholesterol	55	
Visited nutritionist or dietitian in previous year		11	
Medication use			
	Cholesterol lowering	63	
	Blood pressure lowering	58	
	Glucose lowering	8	

BMI, body mass index; SD, standard deviation. More than high school education includes certificate or diploma, bachelors or postgraduate degree. Group, households include living with spouse/partner, family member or friend. Diabetes medical history includes type 1 and type 2 diabetes.

**Table 2:** Comparison of the median/mean daily food group intake (serves/day) estimated for the Aus-SDS1, Aus-SDS2 and the average of the 24-HRs.

Food Group	Aus-SDS1		Aus-SDS2		24-HRs	
	Mean (SD)	Median (IQR)	Mean (SD)	Median (IQR)	Mean (SD)	Median (IQR)
Vegetables	1.53 (0.90)	1.00 (1.00, 2.00)	1.52 (0.90)	1.00 (1.00, 2.00)	1.64 (1.01)	1.42 (1.02, 2.30)
Fruit	1.02 (0.64)	1.00 (0.78, 1.00)	1.12 (0.72)	1.00 (0.78, 1.50)	1.09 (0.77)	0.94 (0.52, 1.58)
Cereal	2.11 (1.05)	2.00 (1.00, 3.00)	2.16 (1.06)	2.00 (0.01, 3.00)	2.32 (1.13)	2.28 (1.59, 3.16)
Legumes and beans	0.20 (0.27)	0.07 (0.02, 0.28)	0.21 (0.32)	0.07 (0.02, 0.28)	0.03 (0.10)	0.00 (0.00, 0.00)
Protein sources *	1.08 (0.63)	1.00 (0.78, 1.00)	1.04 (0.59)	1.00 (0.78, 1.00)	1.14 (0.74)	1.03 (0.61, 1.53)
Dairy sources †	1.03 (0.62)	1.00 (0.78, 1.00)	1.12 (0.72)	1.00 (0.78, 1.50)	1.02 (0.64)	0.92 (0.59, 1.33)

* Protein sources comprised of lean meat and poultry, fish, eggs, tofu, nuts and seeds. † Dairy sources comprised of milk, yoghurt, cheese and alternatives.24-HRs, 24-h recalls; Aus-SDS1, first administered Australian Short Dietary Screener; Aus-SDS2, second administered Australian Short Dietary Screener; IQR, interquartile range; SD, standard deviation.

**Table 3:** Relative validity of the Aus-SDS1, determined by comparison of intakes assessed in Aus-SDS1 and the average of 24-HRs.

Food Group		Comparison of Aus-SDS1 vs. 24-HRs			
	Spearman R (95% CI)	κ Coefficient (95% CI)	Mean Difference * (95% CI) in Intakes between Aus-SDS1 and 24-HRs	*p* Value **	95% LOA
Vegetables	0.82 (0.75, 0.88)	0.58 (0.46, 0.70)	−0.11 (−0.20, −0.01)	0.023	−1.07, 0.85
Fruit	0.64 (0.50, 0.74)	0.44 (0.28, 0.57)	−0.06 (−0.15, 0.02)	0.142	−0.94, 0.81
Cereal	0.84 (0.78, 0.89)	0.51 (0.39, 0.64)	−0.21 (−0.34, −0.08)	0.002	−1.53, 1.11
Legumes and beans	0.82 (0.75, 0.88)	0.12 (0.03, 0.22)	0.17 (0.12, 0.22)	<0.001	−0.32, 0.66
Protein sources †	0.83 (0.75, 0.88)	0.64 (0.51, 0.75)	−0.06 (−0.14, 0.02)	0.118	−0.87, 0.74
Dairy sources ‡	0.71 (0.60, 0.80)	0.44 (0.31, 0.58)	0.01 (−0.07, 0.09)	0.817	−0.83, 0.85

* Values were calculated by subtracting the average of three 24-HR intake from Aus-SDS1 intake. ** *p* value between mean difference in intakes between Aus-SDS1 and 24-HRs, calculated by one-sample *t*-test. † Protein sources comprised of lean meat and poultry, fish, eggs, tofu, nuts and seeds. ‡ Dairy sources comprised of milk, yoghurt, cheese and alternatives. CI, confidence interval; Aus-SDS1, first administered Australian Short Dietary Screener; IQR, interquartile range; κ coefficient, Cohen’s kappa coefficient; LOA, limits of agreement; SD, standard deviation; Spearman r, Spearman’s rank correlation coefficient.

**Table 4:** Reproducibility of Aus-SDS determined by comparison of core food group intake (serves/day) between Aus-SDS1 and Aus-SDS2.

Food Group		Comparison of Aus-SDS1 vs. Aus-SDS2			
	Spearman R (95% CI)	κ Coefficient (95% CI)	Mean Difference * (95% CI) in Intakes between Aus-SDS1 and Aus-SDS2	*p* Value **	95% LOA
Vegetable	0.91 (0.88, 0.94)	0.82 (0.71, 0.90)	−0.02 (−0.09, 0.06)	0.694	−0.78, 0.75
Fruit	0.95 (0.92, 0.96)	0.89 (0.81, 0.97)	0.10 (0.03, 0.16)	0.004	−0.55, 0.74
Cereal	0.84 (0.77, 0.89)	0.69 (0.57, 0.79)	0.05 (−0.07, 0.17)	0.401	−1.13, 1.23
Legumes and beans	0.86 (0.80, 0.90)	0.68 (0.55, 0.80)	0.01 (−0.02, 0.04)	0.501	−0.31, 0.32
Protein sources †	0.86 (0.80, 0.90)	0.68 (0.54, 0.78)	−0.04 (−0.13, 0.06)	0.443	−0.94, 0.87
Dairy sources ‡	0.91 (0.87, 0.94)	0.85 (0.75, 0.93)	0.09 (0.02, 0.17)	0.018	−0.68, 0.86

* Values were calculated by subtracting Aus-SDS2 intake from Aus-SDS1 intake. ** *p* value between mean difference in intakes between Aus-SDS1 and Aus-SDS2, calculated by one-sample *t*-test. † Protein sources comprised of lean meat and poultry, fish, eggs, tofu, nuts and seeds. ‡ Dairy sources comprised of milk, yoghurt, cheese and alternatives.CI, confidence interval; Aus-SDS1 first administered Australian Short Dietary Screener; Aus-SDS2, second administered Australian Short Dietary Screener; IQR, interquartile range; κ coefficient, Cohen’s kappa coefficient; LOA, limits of agreement; SD, standard deviation; Spearman r, Spearman’s rank correlation coefficient.

## Supplementary Materials

The following are available online at https://www.mdpi.com/2072-6643/12/5/1436/s1: Figure S1: Flowchart of study recruitment, Figure S2: Australian Short Dietary Screener (Aus-SDS).

## References

[1] Chang A.Y., Skirbekk V.F., Tyrovolas S., Kassebaum N.J., Dieleman J.L. (2019). Measuring population ageing: An analysis of the Global Burden of Disease Study 2017. Lancet Public Health.

[2] Australian Institute of Health and Welfare (AIHW) (2016). Australia’s Health. http://www.aihw.gov.au/australias-health/2016/.

[3] World Health Organization (2003). Diet, Nutrition, and the Prevention of Chronic Diseases: Report of a Joint WHO/FAO Expert Consultation.

[4] Afshin A., Sur P.J., Fay K.A., Cornaby L., Ferrara G., Salama J.S., Mullany E.C., Abate K.H., Abbafati C., Abebe Z. (2019). Health effects of dietary risks in 195 countries, 1990–2017: A systematic analysis for the Global Burden of Disease Study 2017. Lancet.

[5] Drewnowski A., Shultz J. (2001). Impact of aging on eating behaviors, food choices, nutrition, and health status. J. Nutr. Health Aging.

[6] Senate Community Affairs References Committee (2018). Effectiveness of the Aged Care Quality Assessment and Accreditation Framework for Protecting Residents from Abuse and Poor Practices, and Ensuring Proper Clinical and Medical Care Standards are Maintained and Practised: Interim Report. www.aph.gov.au/Parliamentary_Business/Committees/Senate/Community_Affairs/AgedCareQuality/Interim_report.

[7] Saunders J., Smith T. (2010). Malnutrition: Causes and consequences. Clin. Med. (Northfield Il.).

[8] Tapsell L.C., Neale E.P., Satija A., Hu F.B. (2016). Foods, nutrients, and dietary patterns: Interconnections and implications for dietary guidelines. Adv. Nutr..

[9] Govindaraju T., Sahle B.W., McCaffrey T.A., McNeil J.J., Owen A.J. (2018). Dietary patterns and quality of life in older adults: A systematic review. Nutrients.

[10] Freeland-Graves J.H., Nitzke S. (2013). Position of the academy of nutrition and dietetics: Total diet approach to healthy eating. J. Acad. Nutr. Diet..

[11] National Health & Medical Research Council, Department of Health and Ageing (2013). Eat for Health. Australian Dietary Guidelines: Summary.

[12] Iqbal R., Anand S., Ounpuu S., Islam S., Zhang X., Rangarajan S., Chifamba J., Al-Hinai A., Keltai M., Yusuf S. (2008). Dietary patterns and the risk of acute myocardial infarction in 52 countries. Circulation.

[13] Russell J., Flood V., Rochtchina E., Gopinath B., Allman-Farinelli M., Bauman A., Mitchell P. (2013). Adherence to dietary guidelines and 15-year risk of all-cause mortality. Br. J. Nutr..

[14] Volkert D., Schrader E. (2013). Dietary assessment methods for older persons: What is the best approach?. Curr. Opin. Clin. Nutr. Metab. Care.

[15] Shim J.-S., Oh K., Kim H.C. (2014). Dietary assessment methods in epidemiologic studies. Epidemiol. Health.

[16] Eysteinsdottir T., Thorsdottir I., Gunnarsdottir I., Steingrimsdottir L. (2012). Assessing validity of a short food frequency questionnaire on present dietary intake of elderly Icelanders. Nutr. J..

[17] Thompson F.E., Midthune D., Subar A.F., Kahle L.L., Schatzkin A., Kipnis V. (2004). Performance of a short tool to assess dietary intakes of fruits and vegetables, percentage energy from fat and fibre. Public Health Nutr..

[18] Cook A., Roberts K., O’Leary F., Allman-Farinelli M.A. (2015). Comparison of single questions and brief questionnaire with longer validated food frequency questionnaire to assess adequate fruit and vegetable intake. Nutrition.

[19] Kris-Etherton P., Eissenstat B., Jaax S., Srinath U., Scott L., Rader J., Pearson T. (2001). Validation for MEDFICTS, a dietary assessment instrument for evaluating adherence to total and saturated fat recommendations of the National Cholesterol Education Program Step 1 and Step 2 diets. J. Am. Diet. Assoc..

[20] Blalock S.J., Norton L.L., Patel R.A., Cabral K., Thomas C.L. (2003). Development and assessment of a short instrument for assessing dietary intakes of calcium and vitamin D. J. Am. Pharm. Assoc..

[21] Robinson S., Jameson K., Bloom I., Ntani G., Crozier S., Syddall H., Dennison E., Cooper C., Sayer A. (2017). Development of a short questionnaire to assess diet quality among older community-dwelling adults. J. Nutr. Health Aging.

[22] Bailey R.L., Miller P.E., Mitchell D.C., Hartman T.J., Lawrence F.R., Sempos C.T., Smiciklas-Wright H. (2009). Dietary screening tool identifies nutritional risk in older adults. Am. J. Clin. Nutr..

[23] Bland J.M., Altman D. (1986). Statistical methods for assessing agreement between two methods of clinical measurement. Lancet.

[24] Jackson K.A., Byrne N.M., Magarey A.M., Hills A.P. (2008). Minimizing random error in dietary intakes assessed by 24-h recall, in overweight and obese adults. Eur. J. Clin. Nutr..

[25] Conway J.M., Ingwersen L.A., Moshfegh A.J. (2004). Accuracy of dietary recall using the USDA five-step multiple-pass method in men: An observational validation study. J. Am. Diet. Assoc..

[26] Bliss R.M. (2004). Researchers produce innovation in dietary recall. Agric. Res..

[27] Australian Bureau of Statistics (2010). Australian Health Survey Food model booklet. Canberra, Cat. No. 4363.0.55.001. https://www.ausstats.abs.gov.au/ausstats/subscriber.nsf/0/05E75E65AD98B1C0CA257CD20014B24B/$File/food%20model%20booklet.pdf.

[28] Food Standards Australia New Zealand (2014). AUSNUT 2011–13—Australian Food Composition Database.

[29] Roe M., Bell S., Oseredczuk M., Christensen T., Westenbrink S., Pakkala H., Presser K., Finglas P. (2013). Updated food composition database for nutrient intake. EFSA Supporting Publ..

[30] Australian Bureau of Statistics Australian Health Survey: Users’ Guide, 2011–2013, Discretionary Foods, Cat. No. 4363.0.55.001. http://www.abs.gov.au/ausstats/abs@.nsf/Lookup/4363.0.55.001Chapter65062011-13.

[31] Giles G.G., Ireland P.D. (1996). Dietary Questionnaire for Epidemiological Studies (Version 3.2).

[32] Giavarina D. (2015). Understanding bland altman analysis. Biochem. Med..

[33] Lei L., Rangan A., Flood V.M., Louie J.C.Y. (2016). Dietary intake and food sources of added sugar in the Australian population. Br. J. Nutr..

[34] Pomerleau J., Lock K., Knai C., McKee M. (2005). Effectiveness of Interventions and Programmes Promoting Fruit and Vegetable Intake.

[35] Masson L.F., MCNeill G., Tomany J., Simpson J., Peace H.S., Wei L., Grubb D., Bolton-Smith C. (2003). Statistical approaches for assessing the relative validity of a food-frequency questionnaire: Use of correlation coefficients and the kappa statistic. Public Health Nutr..

[36] Shatenstein B., Payette H. (2015). Evaluation of the relative validity of the short diet questionnaire for assessing usual consumption frequencies of selected nutrients and foods. Nutrients.

[37] Pisani P., Faggiano F., Krogh V., Palli D., Vineis P., Berrino F. (1997). Relative validity and reproducibility of a food frequency dietary questionnaire for use in the Italian EPIC centres. Int. J. Epidemiol..

[38] Whitton C., Ho J.C.Y., Rebello S.A., van Dam R.M. (2018). Relative validity and reproducibility of dietary quality scores from a short diet screener in a multi-ethnic Asian population. Public Health Nutr..

[39] Ibiebele T.I., Parekh S., Mallitt K.-a., Hughes M.C., O’Rourke P.K., Webb P.M. (2009). Reproducibility of food and nutrient intake estimates using a semi-quantitative FFQ in Australian adults. Public Health Nutr..

[40] Rolstad S., Adler J., Rydén A. (2011). Response burden and questionnaire length: Is shorter better? A review and meta-analysis. Value Health.

[41] Tonkin E., Kennedy D., Golley R., Byrne R., Rohit A., Kearns T., Hanieh S., Biggs B.-A., Brimblecombe J. (2018). The relative validity of the menzies remote short-item dietary assessment tool (MRSDAT) in aboriginal Australian children aged 6–36 months. Nutrients.

[42] Bartholome L.T., Peterson R.E., Raatz S.K., Raymond N.C. (2013). A comparison of the accuracy of self-reported intake with measured intake of a laboratory overeating episode in overweight and obese women with and without binge eating disorder. Eur. J. Nutr..

[43] Manjer J., Carlsson S., Elmståhl S., Gullberg B., Janzon L., Lindström M., Mattisson I., Berglund G. (2001). The Malmö Diet and Cancer Study: Representativity, cancer incidence and mortality in participants and non-participants. Eur. J. Cancer Prev..

[44] Dijkstra W., Smit J.H., Comijs H.C. (2001). Using social desirability scales in research among the elderly. Qual. Quant..

[45] Shahar D., Yerushalmi N., Lubin F., Froom P., Shahar A., Kristal-Boneh E. (2001). Seasonal variations in dietary intake affect the consistency of dietary assessment. Eur. J. Epidemiol..

[46] De la Fuente-Arrillaga C., Ruiz Z.V., Bes-Rastrollo M., Sampson L., Martinez-Gonzalez M.A. (2010). Reproducibility of an FFQ validated in Spain. Public Health Nutr..

[47] Vereecken C.A., Maes L. (2003). A Belgian study on the reliability and relative validity of the Health Behaviour in School-Aged Children food-frequency questionnaire. Public Health Nutr..

