# Supplementary material for: Development, Relative Validity and Reproducibility of the Aus-SDS (Australian Short Dietary Screener) in Adults Aged 70 Years and above
Source: Nutrients. 2020 May 15;12(5):1436. doi: 10.3390/nu12051436 (PMC7284974; doi:10.3390/nu12051436)
Supplement: Supplementary file 1 [file nutrients-12-01436-s001.pdf]

Supplementary Figure S1: Flowchart of study recruitment

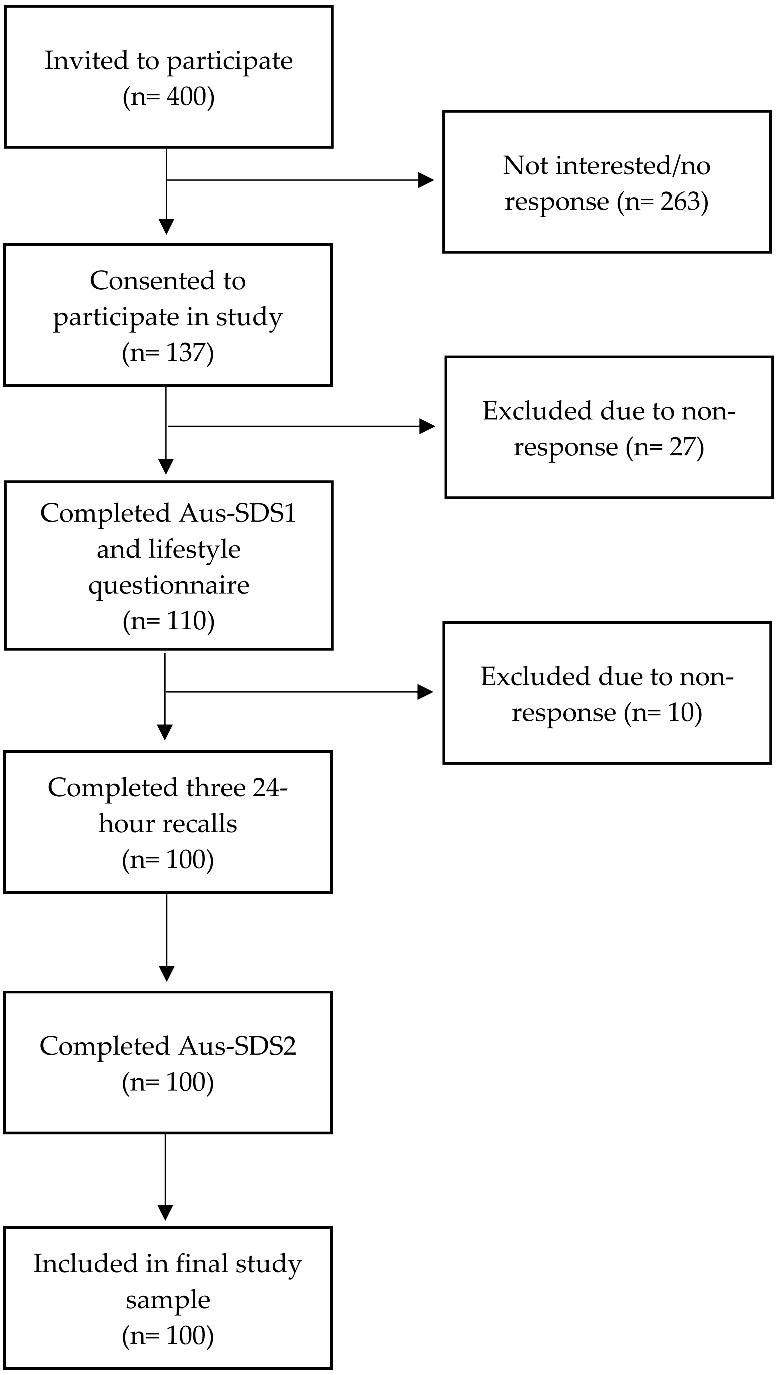

ID

Australian Short Dietary Screener (Aus-SDS)

Please complete all pages in black or blue biro pens ONLY. Anything else, such as pencil or felt-tip, does not read clearly.

To answer a question, place either a number or cross inside the box like this: X or 3

If you make a mistake, DO NOT use Liquid Paper. Instead, place a diagonal line through the incorrect answer, then put a cross in the box of your preferred response.

This screening tool asks about your usual dietary intake of the five key food groups: vegetables, fruit, grains, meat and protein sources and dairy and dairy alternatives.

It can be surprisingly difficult to remember what we eat, as it often changes from day-to-day and with the seasons, but we ask that you estimate what you have usually or typically eaten over the past year. Please try to remember of your dietary intake of all key food groups, remembering they may be included in mixed meals.

Today's date: ☐☐/☐☐/☐☐☐☐ (Day / Month / Year)

Please think about your usual intake over the past 12 MONTHS. Please mark **ONE** response only for each question

Question 1: How many serves of each did you usually eat **PER DAY**?

	**NEVER**	**Less than 1** serve per day	**1** serve per day	**2** serves per day	**3** serves per day	**4** serves per day	**5 or more** serves per day
**VEGETABLES** Count 1 serve as either; 1 cup of raw salad vegetables, or 1/2 cup of cooked vegetables.	☐	☐	☐	☐	☐	☐	☐

Examples
Green vegetables: broccoli, brussel sprouts, bok choy, cabbages, cauliflower, kale lettuce, silver beet, spinach, snow peas.
Root/tuber/bulb vegetables: potato, carrots, beetroot, onions, garlic, swede, turnip, shoots.
Other vegetables: tomato, celery, cucumber, zucchini, squash, avocado, eggplant, capsicum, pumpkin, mushroom.

If **LESS THAN 1 SERVE PER DAY**, how many serves did you usually eat?

**Less than 1** serve per **month**	**1-3** serves per **month**	**1** serve per **week**	**2** serves per **week**	**3-4** serves per **week**	**5-6** serves per **week**
☐	☐	☐	☐	☐	☐

	NEVER	**Less than 1** serve per day	**1** serve per day	**2** serves per day	**3** serves per day	**4** serves per day	**5 or more** serves per day
**FRUIT**<br>Count 1 serve as either; a whole piece of fruit such as an apple, banana or orange, or a handful of smaller fruit such as grapes, berries or sultanas, or a cup of fruit salad or stewed fruit.<br><br>**Examples**<br>**Pome fruits**: apple, pear.<br>**Citrus fruits**: orange, mandarin, grapefruit.<br>**Stone fruits**: apricot, cherry, peach, nectarine, plum.<br>**Tropical fruits**: banana, paw paw, mango, pineapple,melon.<br>**Berries**: strawberry, blueberry, blackberry, raspberry, cranberry.<br>**Other fruits**: grapes, passionfruit.<br>**Dried fruit**: sultanas, dried apricots, dried cranberries, dried dates, dried prunes.	☐	☐	☐	☐	☐	☐	☐

If **LESS THAN 1 SERVE PER DAY**, how many serves did you usually eat?

**Less than 1** serve per **month**	**1-3** serves per **month**	**1** serve per **week**	**2** serves per **week**	**3-4** serves per **week**	**5-6** serves per **week**
☐	☐	☐	☐	☐	☐

	NEVER	**Less than 1** serve per day	**1** serve per day	**2** serves per day	**3** serves per day	**4** serves per day	**5 or more** serves per day
**GRAINS**<br>Count 1 serve as either; 1 slice of bread, 1/2 cup of cooked grains, pasta and couscous, 1 crumpet or scone, 1/2 cup cooked porridge, 2/3 cup wheat cereal flakes.<br><br>**Examples**<br>**Bread**: wholemeal, wholegrain, white, rye, pita, lavash, naan, focaccia, crispbreads.<br>**Breakfast cereals**: ready to eat, high fibre (wholegrain) oats, porridge, muesli, wheat-bix.<br>**Grains**: rice, barley, corn, polenta, buckwheat, spelt, millet, sorghum, triticale, rye, quinoa, semolina.<br>**Other cereal products**: pasta, noodles, rice cakes, couscous, popcorn.	☐	☐	☐	☐	☐	☐	☐

If **LESS THAN 1 SERVE PER DAY**, how many serves did you usually eat?

**Less than 1** serve per **month**	**1-3** serves per **month**	**1** serve per **week**	**2** serves per **week**	**3-4** serves per **week**	**5-6** serves per **week**
☐	☐	☐	☐	☐	☐

	NEVER	**Less than 1** serve per day	**1** serve per day	**2** serves per day	**3** serves per day	**4** serves per day	**5 or more** serves per day
**LEGUME and BEANS**<br>Count 1 serve as either; 1/2 cup cooked or canned legumes/beans, 1/2 cup tofu.<br>**Examples**<br>Red kidney beans, soybeans, lima beans, cannellini beans, baked beans, chickpeas, lentils, split peas, tofu.	☐	☐	☐	☐	☐	☐	☐

If **LESS THAN 1 SERVE PER DAY**, how many serves did you usually eat?

**Less than 1** serve per **month**	**1-3** serves per **month**	**1** serve per **week**	**2** serves per **week**	**3-4** serves per **week**	**5-6** serves per **week**
☐	☐	☐	☐	☐	☐

	NEVER	**Less than 1** serve per day	**1** serve per day	**2** serves per day	**3** serves per day	**4** serves per day	**5 or more** serves per day
**MEAT and other PROTEIN SOURCES**<br>Count 1 serve as either; lean red meat (e.g. 1 small steak), lean poultry (e.g. 1 small chicken breast), fish fillet (e.g. 1 medium fill fillet), one small can of fish, 2 large eggs.<br>**Examples**<br>**Lean meats**: beef, lamb, veal, pork, kangaroo, lean lower salt sausages.<br>**Poultry**: chicken, turkey, duck, emu, goose, bush birds.<br>**Fish and seafood**: fish, prawns, crab, lobster, mussels, oysters, scallops, clams.<br>**Eggs**: chicken, duck eggs.<br>**Nuts and seeds**: almonds, pine nuts, walnuts, macadamia, hazelnut, cashew, peanut, nut spreads, pumpkin seeds, sesame seeds, sunflower seeds, brazil nuts.	☐	☐	☐	☐	☐	☐	☐

If **LESS THAN 1 SERVE PER DAY**, how many serves did you usually eat?

**Less than 1** serve per **month**	**1-3** serves per **month**	**1** serve per **week**	**2** serves per **week**	**3-4** serves per **week**	**5-6** serves per **week**
☐	☐	☐	☐	☐	☐

	NEVER	**Less than 1** serve per day	**1** serve per day	**2** serves per day	**3** serves per day	**4** serves per day	**5 or more** serves per day
**MILK, YOGHURT, CHEESE and ALTERNATIVES**<br>Count 1 serve as either; 1 cup fresh UHT long life, powdered, buttermilk, soy or rice drink, 1/2 cup evaporated milk, 2 slices of hard cheese such as cheddar, 3/4 cup yoghurt.<br><br>**Examples**<br>**Milks**: all reduced fat or full cream milks, plain and flavoured, long life milks, powdered milks, evaporated milks, soy beverages.<br>**Yoghurt**: all yoghurt including reduced fat and full cream, plain and flavoured, soy yoghurt.<br>**Cheese**: all hard cheeses and soy cheeses, reduced or full fat.	☐	☐	☐	☐	☐	☐	☐

If **LESS THAN 1 SERVE PER DAY**, how many serves did you usually eat?

**Less than 1** serve per **month**	**1-3** serves per **month**	**1** serve per **week**	**2** serves per **week**	**3-4** serves per **week**	**5-6** serves per **week**
☐	☐	☐	☐	☐	☐
